# The FAD synthetase from the human pathogen *Streptococcus pneumoniae*: a bifunctional enzyme exhibiting activity-dependent redox requirements

**DOI:** 10.1038/s41598-017-07716-5

**Published:** 2017-08-08

**Authors:** María Sebastián, Erandi Lira-Navarrete, Ana Serrano, Carlos Marcuello, Adrián Velázquez-Campoy, Anabel Lostao, Ramón Hurtado-Guerrero, Milagros Medina, Marta Martínez-Júlvez

**Affiliations:** 1 Departamento de Bioquímica y Biología Molecular y Celular, Facultad de Ciencias, Universidad de Zaragoza, Zaragoza, Spain; 2 Instituto de Biocomputación y Física de Sistemas Complejos (BIFI) and GBsC-CSIC and BIFI-CSIC Joint Units, Universidad de Zaragoza, Zaragoza, Spain; 3 Laboratorio de Microscopías Avanzadas (LMA), Instituto de Nanociencia de Aragón (INA), and Fundación INA, Universidad de Zaragoza, Zaragoza, Spain; 4 Fundación ARAID, Diputación General de Aragón, Aragón, Spain; 5 Aragon Institute for Health Research (IIS Aragon), Zaragoza, 50009 Spain; 6 Copenhagen Center for Glycomics, Department of Cellular and Molecular Medicine, School of Dentistry, University of Copenhagen, Copenhagen, DK-2200 Denmark; 7 Centro de Investigaciones Biológicas, CSIC, Ramiro de Maeztu 9, E-28040 Madrid, Spain; 8 Univ Reims, Lab Rech Nanosci, EA4682, F-51100 Reims and INRA, FARE Lab, F-51100 Reims, France

## Abstract

Prokaryotic bifunctional FAD synthetases (FADSs) catalyze the biosynthesis of FMN and FAD, whereas in eukaryotes two enzymes are required for the same purpose. FMN and FAD are key cofactors to maintain the flavoproteome homeostasis in all type of organisms. Here we shed light to the properties of the hitherto unstudied bacterial FADS from the human pathogen *Streptococcus pneumoniae* (*Spn*FADS). As other members of the family, *Spn*FADS catalyzes the three typical activities of prokaryotic FADSs: riboflavin kinase (RFK), ATP:FMN:adenylyltransferase (FMNAT), and FAD pyrophosphorylase (FADpp). However, several *Spn*FADS biophysical properties differ from those of other family members. In particular; i) the RFK activity is not inhibited by the riboflavin (RF) substrate, ii) the FMNAT and FADSpp activities require flavin substrates in the reduced state, iii) binding of adenine nucleotide ligands is required for the binding of flavinic substrates/products and iv) the monomer is the preferred state. Collectively, our results add interesting mechanistic differences among the few prokaryotic bifunctional FADSs already characterized, which might reflect the adaptation of the enzyme to relatively different environments. In a health point of view, differences among FADS family members provide us with a framework to design selective compounds targeting these enzymes for the treatment of diverse infectious diseases.

## Introduction

Prokaryotic FAD synthetases (FADSs) are bifunctional and bimodular enzymes that first catalyze the synthesis of flavin mononucleotide (FMN) from riboflavin (RF, vitamin B_2_) through an ATP:riboflavin kinase activity (RFK, EC 2.7.1.26) and subsequently the adenylylation of FMN to produce flavin adenine dinucleotide (FAD) using an ATP:FMN:adenylyltransferase activity (FMNAT, EC 2.7.7.2)^1–3^. For some of them, the FMNAT activity has also been shown to be reversible, exhibiting therefore a FAD pyrophosphorylase activity (FADpp)^4, 5^. In mammals and yeasts two independent enzymes catalyze these reactions^6–9^. Eukaryotic RFKs exhibit structural homology with the C-terminal module of prokaryotic bifunctional FADSs^10–12^. On the contrary eukaryotic FMNATs do not exhibit similarity to the prokaryotic N-terminal modules, which belong to the nucleotidyltransferase superfamily^13–15^. Since FMN and FAD are essential and versatile cofactors in a plethora of vital redox processes^16–20^, and also participate in signal transduction during apoptosis^21^, embryonic development^22^, chromatin remodeling^23^, nucleotide synthesis^24^, ^t^RNA methylation^25^, protein folding^26^, and light dependent processes in plants as well as in sensory transduction in microorganisms^27–31^, their biosynthesis is worth to be understood.

Structures of prokaryotic FADSs are available only for *Thermotoga maritima* (*Tm*FADS, PDB ID: 1MRZ,^11, 32^), *Corynebacterium ammoniagenes* (*Ca*FADS, PDB ID: 2X0K^10^) and the human pathogen *Streptococcus pneumoniae* (*Spn*FADS, PDB ID: 3OP1). Except for the exhaustive biochemical and biophysical characterization of *Ca*FADS^1, 5, 33^, the rest of family members have been poorly studied^4, 34, 35^. *Spn*FADS shows the typical sequence and structural features of prokaryotic bifunctional FADSs, and as such it would be predicted to contain RFK, FMNAT and FADpp activities (Supplementary Fig. S1; r.m.s.d. of 1.63 Å encompassing 247 residues and 30% sequence identity with *Ca*FADS). The N-terminal module (residues 1–186) is an α/β dinucleotide binding domain that by homology to other family members is expected to catalyze the FMNAT and FADpp activities^10, 11^. Likewise, the C-terminal module (residues 187–305), predicted to catalyze the RFK activity^10, 36^, consists of a β barrel formed by six antiparallel β-strands and a long terminal α-helix. The different structures together with the significant differences at the catalytic centers between the N-terminal modules of bifunctional FADSs and the mammalian FMNAT enzymes^10, 14^ make prokaryotic FADSs attractive exploitable targets for the treatment of human infectious diseases^37^. In this context, *Spn*FADS might be considered as a drug target for *S*. *pneumoniae*, a pathogen almost exclusive for humans, that is the leading cause of invasive bacterial pneumoniae disease in children, in the elderly and in immunodepressed patients^38–40^.

We present here the biochemical and biophysical characterization of *Spn*FADS, the only FADS from a pathogenic prokaryote whose structure is available. Our data indicate that *Spn*FADS catalyzes the RFK, FMNAT and FADpp activities. However, it differs from other family members in two major catalytic aspects: its RFK activity does not exhibit inhibition by the RF substrate and the FMNAT activity requires the flavin substrate in its reduced state for substrate binding and catalysis. In addition, the prior binding of adenine nucleotides is required for the recognition of flavins, and the enzyme exits mainly in the monomeric state during catalysis. These results exemplify how the same type of enzymes from different organisms has evolved different strategies to perform the same reaction, presumably due to their unique environment. Our data also point to these bacterial enzymes as promising pharmacological targets because not only differ between themselves but also from the human enzyme.

## Results

### *Spn*FADS is purified free of flavin ligands

Typically, the yield after purification was 3.5 mg of *Spn*FADS *per* liter of *E*.*coli* culture. Enriched *Spn*FADS fractions along purification did not show notorious absorbance at 450 nm, indicating the purified protein does not contain any flavin bound under these conditions. Thus, the final absorption spectrum of purified *Spn*FADS exhibits a single peak at 279 nm (Supplementary Fig. S2) and in contrast to *Ca*FADS^13^, *Spn*FADS does not co-purify with FAD, suggesting a much lower affinity for the oxidized flavinic substrates/products. The far-ultraviolet (UV) circular dichroism (CD) spectrum of *Spn*FADS exhibits a negative band at 208 nm typical of secondary α-helix structure and a slightly less intense negative band around 222 nm (Supplementary Fig. S2). Analysis of the far-UV CD spectrum using the method of Raussens^41^ predicted 28.3, 19.9, 12.5 and 34.5% of content in α helix, ß strand, turn and random, respectively, in agreement with its 3D-structure containing 29% α helix and 28% β strand (Supplementary Fig. S1). Near-UV CD spectrum of *Spn*FADS shows a broad 265–290 nm negative band with minima at 280.5 and 287 nm related with the protein tertiary structure organization (Supplementary Fig. S2). Altogether these data indicate that the purified protein is correctly folded.

### *Spn*FADS catalyzes the RFK, FMNAT and FADpp activities

**Figure 1:**
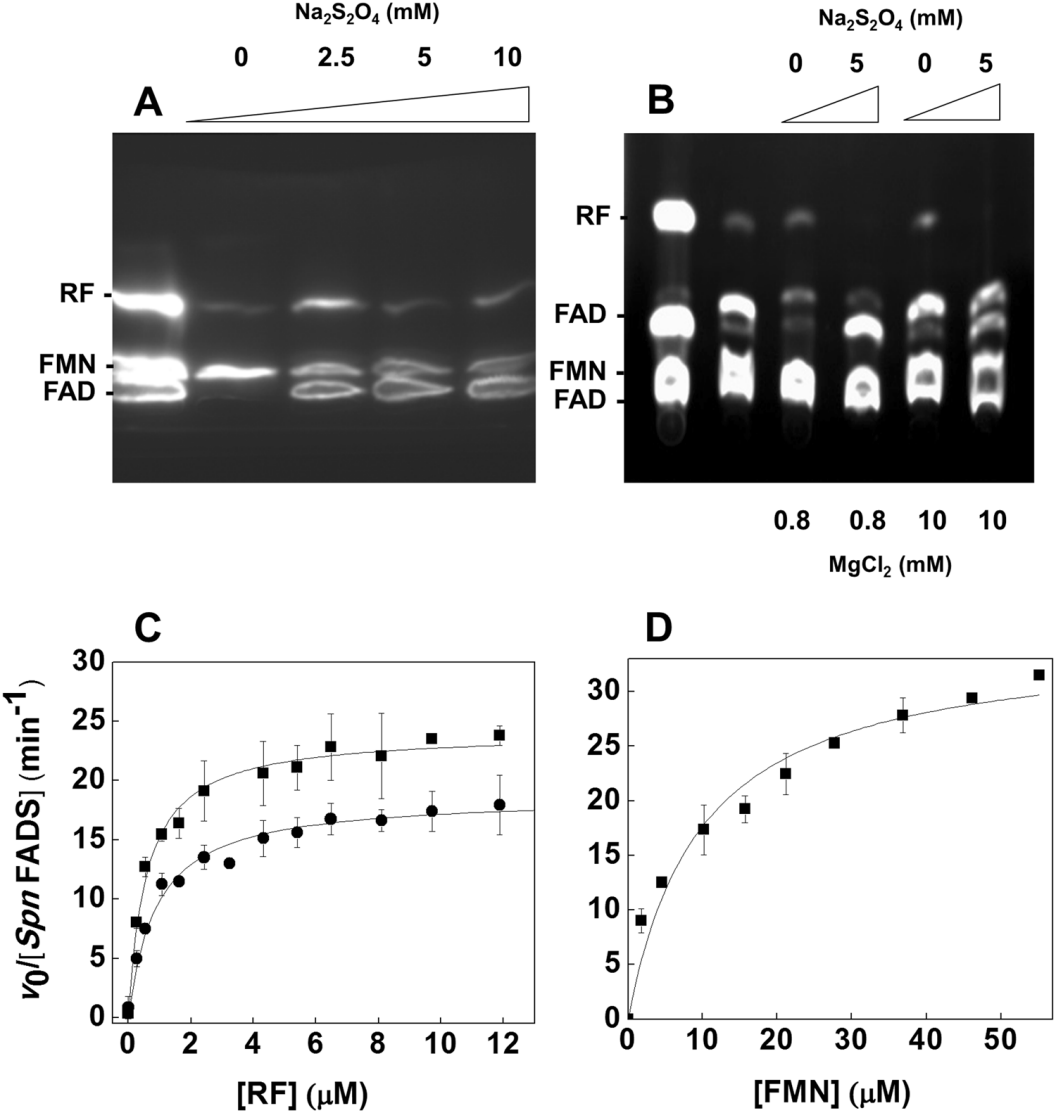
The RFK, FMNAT and FADpp activities of *Spn*FADS. (**A**) Thin layer chromatography of the products of the RFK and FMNAT activities. Reaction mixtures contained around 500 nM *Spn*FADS, 50 μM RF, 0.2 mM ATP, and several concentrations of sodium dithionite. First lane to the left corresponds to a standard solution containing RF, FMN and FAD at 50 μM each. (**B**) Thin layer chromatography of the products of FADpp activity. Reaction mixtures contained around 500 nM *Spn*FADS, 50 μM FAD, 0.2 mM PPi, and several concentrations of sodium dithionite. First lane corresponds to the standard solutions and second line to a free FAD sample with 10 mM MgCl_2_. (**C**) Rates for RFK activity as a function of the RF concentration at saturating ATP (412 μM) without (●) and with 2.6 mM (■) sodium dithionite. (**D**) Rates for the FMNAT activity as a function of the FMN concentration at saturating ATP (252 μM) and 3 mM sodium dithionite. Unless otherwise stated all measurements were carried out at 25 °C in 20 mM PIPES, pH 7.0, with 0.8 mM MgCl_2_.

A qualitative assay was initially performed to evaluate whether *Spn*FADS was able to catalyze the RFK, FMNAT and FADpp activities by resolving the flavin reaction products by thin layer chromatography (TLC). As shown in Fig. 1A, *Spn*FADS catalyzes the conversion of RF into FMN, both in the absence and in the presence of a reducing agent. However, reducing conditions (2.5 mM of sodium dithionite) are required for the transformation of FMN into FAD (Fig. 1A), as well as of FAD into FMN (Fig. 1B).

### The reduced state of flavins is critical for the FMNAT activity but not for the RFK one

Prior to determining the kinetic parameters for the RFK activity of *Spn*FADS, we quantitatively evaluated the influence of the reducing environment (sodium dithionite) as well as of Mg^2+^ on this reaction. Under saturating concentrations of substrates (RF and ATP) rates for RF transformation were independent on the reducing agent concentration (Supplementary Fig. S3), indicating that the *Spn*FADS RFK activity does not depend on its redox environment. On the contrary, Mg^2+^ enhances the RFK activity, being the rate of the process practically constant at cation concentrations over 0.4 mM (Supplementary Fig. S3). Thus, herein a concentration of 0.8 mM MgCl_2_ was used. Steady-state rates for the RFK activity showed saturation profiles for both substrates (Fig. 1C), contrary to *Ca*FADS that has strong inhibition by excess of RF^5, 33^. Thus, profiles for the RFK activity of *Spn*FADS were fit to the Michaelis-Menten model, allowing determination of *k*_cat_, *K*_m_^RF^ and *K*_m_^ATP^ values of 18.7 min^−1^, 0.9 µM and 54.0 µM, respectively. These values were only slightly influenced by the presence of the reducing agent (Table 1, Fig. 1C).

**Table 1:** Steady-state kinetic parameters for the RFK and FMNAT activities of *Spn*FADS.

RFK activity	*Reductant*	*k*_cat_ (min^−1^)	*K*_m_^RF^ (μM)	*k*_cat/_ *K*_m_^RF^ (min^−1^ μM^−1^)	*k*_cat_ (min^−1^)	*K*_m_^ATP^ (μM)	*k*_cat/_ *K*_m_^ATP^ (min^−1^ μM^−1^)
*Spn*FADS	−	18.7	0.9	21.0	19.5^a^	54.0	0.4
	+	24.1	0.6	41.5	13.4^a^	34.1	0.4
*Ca*FADS^b,c^	−	< 302	< 13	23.2	68	13.7	4.9
*Lm*FADSI^d^	+	0.40	6.9	0.057			
*Hs*RFK^f^	+	0.50	36	0.014			
*Bs*FADS^g^	+	0.70	55	0.013			
*Sd*FADS^g^	+	0.30	40	0.007			
*Ec*FADS^h^	+	0.39	2	0.20			
FMNAT activity		***k***_**cat**_ (**min** ^**−1**^)	***K***_**m**_^**FMN**^ (**µM**)	***k***_**cat**/_ ***K***_**m**_^**FMN**^ (**min** ^**−1**^ **µM** ^**−1**^)	***k***_**cat**_ (**min** ^**−1**^)	***K***_**m**_^**ATP**^ (**µM**)	***k***_**cat**/_ ***K***_**m**_^**ATP**^ (**min** ^**−1**^ **µM** ^**−1**^)
*Spn*FADS	+	34.9	9.8	3.6	23.2^e^	31.6	0.7
*Ca*FADS^b^	−	17.0	1.2	14.3	17.0	35.7	0.5
*Lm*FADSI^d^	+	4.39	29.2	0.15			
*Lm*FADSII^d^	+	0.51	12.9	0.04			
*Ec*FADS^h^	+	0.06	4	0.015			

Parameters obtained at 25 °C in 20 mM PIPES, pH 7.0, 0.8 mM MgCl_2_ and, when indicated 3 mM sodium dithionite as reductant. Estimated errors in *k*_cat_ and *K*_m_ are within ± 10%.

^a^ *k*_cat_ measured at 6 μM of RF. ^b^Data from^33^. Data were obtained at 25 °C in 20 mM PIPES, pH 7.0 and 0.8 mM and 10 mM MgCl_2_, respectively, for the RFK and FMNAT activities. ^c^Inhibition by substrate prevented the determination of true parameters and these correspond to apparent constants, *k*_cat_, _app_, *K*_m_, _app_^RF^, *K*_m_,_app_^ATP^, *k*_cat_,_app_/*K*_m_,_app_^RF^ and *k*_cat_,_app_/*K*_m_,_app_^ATP^. ^d^Data from^35^. Data obtained in 50 mM potassium phosphate, pH 7.5. ^e^ *k*_cat_ measured at 27.7 µM of FMN. ^f^Data from^42^. Data obtained in 50 mM potassium phosphate, pH 7.5. ^g^Data from^34^. Data obtained in 100 mM potassium phosphate, pH 7.5. ^h^FADS from *Escherichia coli*. Data from^43^. Data obtained in 50 mM potassium phosphate, pH 7.5.

The FMNAT activity of *Spn*FADS was determined following the transformation of FMN into FAD. We again evaluated first the influence of Mg^2+^ concentration and of reducing conditions in the FMNAT activity. The presence of sodium dithionite was a requirement for the transformation of FMN into FAD (Supplementary Fig. S3), being the rate of the process constant over 2 mM sodium dithionite. On the contrary, the FMNAT activity was very poor in the presence of milder reducing agents as DTT (not shown), suggesting that strong reducing conditions are required to achieve catalysis. Maximal activity was observed in the range of 0.8–1.2 mM of the divalent cation (Supplementary Fig. S3), being the observed rates in its absence up to 5-fold lower. Thus, 0.8 mM MgCl_2_ and 3 mM sodium dithionite were selected to further characterize the FMNAT kinetic parameters of *Spn*FADS. Observed rates exhibited Michaelis-Menten saturation profiles (Fig. 1D) with *k*_cat_, *K*_m_^FMN^ and *K*_m_^ATP^ values of 34.9 min^−1^, 9.8 µM and 31.6 µM, respectively (Table 1). The need of reducing conditions for the FMNAT activity suggests that either the isoalloxazine ring of FMN needs to be reduced for the reaction to occur, or the reaction depends on a conformational change of the flavin binding site of the FMNAT module of *Spn*FADS induced by the redox environment. To clarify this matter an additional experiment was performed. The FMNAT activity of *Spn*FADS was measured under anaerobic conditions, but in the absence of dithionite, using as substrate the photo-reduced FMN. HPLC analysis of the reaction products revealed that *Spn*FADS is able to transform FMN into FAD in the samples containing photo-reduced FMN in contrast with the negative controls where FMN is oxidized. Therefore, we conclude that FMN needs to be in its reduced form to get adenylylated by *Spn*FADS.

### *Spn*FADS does not bind oxidized flavins by itself

**Figure 2:**
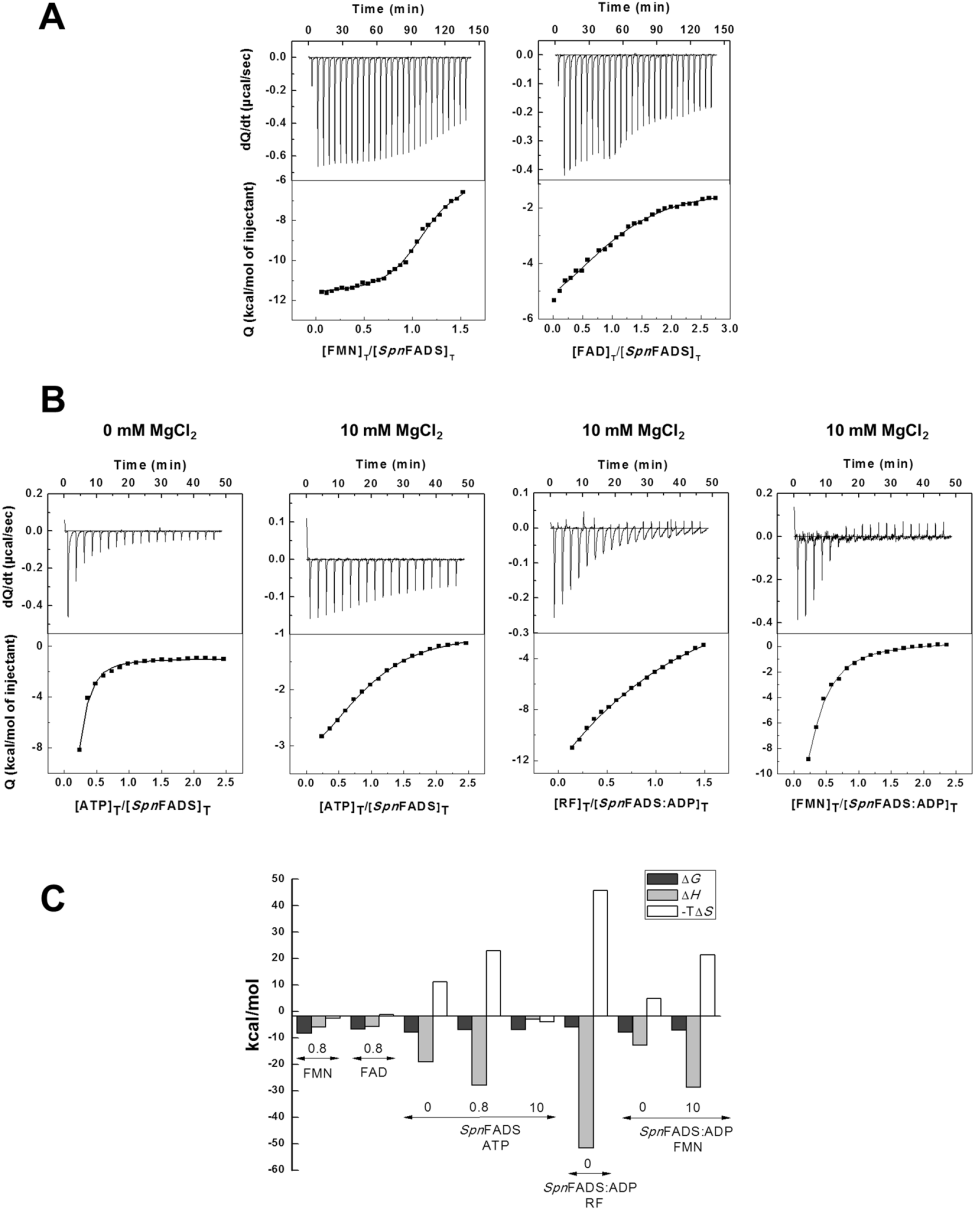
Binding of ligands to *Spn*FADS. (**A**) Calorimetric titrations of *SpnF*ADS with reduced FMN and FAD, performed at 20 °C in 20 mM PIPES, pH 7.0, 0.8 mM MgCl_2_, 4 mM sodium dithionite, 10 mM glucose, 0.5 U/mL glucose oxidase (degassed). (**B**) Calorimetric titrations of *SpnF*ADS with ATP and of the preformed *Spn*FADS:ADP complex with RF or FMN, at 20 °C in 20 mM PIPES, pH 7.0, and at the indicated concentrations of MgCl_2_. In all titration figures upper panels show the thermograms for the interactions and the lower panels show the corresponding binding isotherms with integrated normalized heats. (**C**) Thermodynamic dissections of the interaction of *Spn*FADS with reduced FAD, reduced FMN and ATP, as well as of the preformed *Spn*FADS:ADP complex with RF and FMN. The binding Gibbs energy (Δ*G*), enthalpy (Δ*H*), and entropy (-TΔ*S*) are represented in dark grey, light grey and white bars, respectively. MgCl_2_ concentrations in mM used in each experiment are indicated by numbers.

Isothermal titration calorimetry (ITC) thermograms for the titration of *Spn*FADS with oxidized RF, FMN or FAD, either in presence or absence of Mg^2+^, indicated that under the assayed aerobic conditions none of these flavins binds to the protein with detectable enthalpic contribution (Supplementary Fig. S4). Therefore, we also titrated *Spn*FADS with FMN and FAD in a flavin reducing buffer and in the presence of 0.8 mM Mg^2+^. As shown in Fig. 2A, under such conditions binding of the reduced forms of FMN and FAD to the enzyme was detected. Both reduced cofactors bind to a single *Spn*FADS binding site (Fig. 2A and Table 2). *K*_d_^FMN^ is in the low micromolar range, and binding of reduced FAD is 15-fold weaker. Enthalpic contribution to the binding are very similar for both flavins, but the entropic contributions favor FMN binding (Fig. 2C). These results thus indicate that the FMNAT/FADpp sites of *Spn*FADS only bind the reduced states of FMN or FAD.

**Table 2:** Thermodynamic parameters for the interaction of *Spn*FADS with the substrates and products of its activities obtained by ITC.

Receptor	Ligand	MgCl_2_ (mM)	*K*_d_ (μM)	Δ*G* (kcal/mol)	Δ*H* (kcal/mol)	−TΔ*S* (kcal/mol)	N (binding stoichiometry)
*Spn*FADS	ATP	0	1.7	−7.9	−19.1	11.2	∼1
		0.8	9.4	−6.9	−27.8	23.0	∼1
		10	9.2^b^	−6.9^b^	−3.0^b^	−3.9^b^	∼2
	ADP	0	4.1	−7.3	−12.9	5.5	∼1
		10	30.0	−6.2	−19.4	13.3	∼1
	RF, FMN, FAD	0	n.d.	n.d.	n.d.	n.d.	n.d.
		10					
	FMN^a^	0.8	0.8	−8.3	−5.8	−2.5	∼1
	FAD^a^	0.8	12.5	−6.7	−5.6	−1.1	∼1
*Spn*FADS:ADP	RF	0	0.3	−9.0	−17.2	8.2	∼1
		10	51.3^b^	−5.9^b^	−51.6^b^	45.7^b^	∼2
	FMN	0	1.5	−7.9	−12.8	4.9	< 1
		10	5.9	−7.1	−28.6	21.5	< 1
*Spn*FAD:PP_i_	FAD	0	n.d.	n.d.	n.d.	n.d.	n.d.

Data obtained at 20 °C in 20 mM PIPES, pH 7.0. Estimated errors in *K*_d_ are ± 20% and ± 0.3 kcal/mol in Δ*H* and –TΔ*S*.

n.d. No enthalpic change was detected along the titration. ^a^Measurements performed in the presence of 4 mM sodium dithionite, 10 mM glucose and 0.5 U/ml glucose oxidase. ^b^Data corresponding to average values of two similar and independent binding sites (*K*_d,av_, Δ*G*_av_, Δ*H*_av_, −TΔ*S*_av_).

Thermograms for the titration of *Spn*FADS with ATP indicated a single binding site for this nucleotide (presumably at the FMNAT site as in *Ca*FADS), both in the absence and presence of 0.8 mM of Mg^2+^ (Fig. 2B and Table 2), although binding affinities and interaction parameters differ between these two conditions. Thus, ATP and ATP:Mg^2+^ allocate differently into the protein binding site, suggesting that the ion contributes to the nucleotide-protein-bound conformation. Titrations at higher Mg^2+^ concentrations showed two independent ATP binding sites, putatively related with each catalytic site, with similar affinities between them, and also similar to that of the site found at lower Mg^2+^ concentrations. However, enthalpic and entropic contributions to the ATP binding result highly influenced by Mg^2+^; in its absence, and at low concentrations, the nucleotide binding is enthalpically driven with an opposing entropic contribution, while at high cation concentrations relatively mild entropic and enthalpic contributions favor the binding (Fig. 2C). Thermodynamic parameters for the *Spn*FADS titration with ADP also differ depending on Mg^2+^ concentration. In the absence of the cation, a single high affinity binding site is observed, being the binding driven by a favorable enthalpic contribution. At 10 mM of Mg^2+^, a second binding site appears with lower affinity for the nucleotide, being the averaged *K*_d_ (*K*_d,av_) at least 7-times higher. ADP binding in the presence of 10 mM Mg^2+^ is also enthalpically driven, but in this case the enthalpic contribution is much higher, being the entropic term mightily unfavorable. Thus high Mg^2+^ concentrations induce the generation of a second binding site for the adenine nucleotide ligands, both ATP and ADP, and influence the way the ligands allocate into the active site, as also supported by the different thermodynamic contributions to free energy of the binding.

Thermograms for the titration of mixtures containing *Spn*FADS plus ADP with oxidized RF or FMN showed that the presence of the adenine nucleotide promotes binding of both of these flavins (Fig. 2 and Table 2). Under these conditions RF in presence of 10 mM MgCl_2_, apparently binds at two independent binding sites with moderate affinity, *K*_d,av_ 51.3 µM, being the averaged binding enthalpically driven and the entropic contribution highly unfavorable. However, in the absence of Mg^2+^ only one binding site with strong affinity is detected. By contrast FMN binds at a single site with stronger affinity than those found for RF, being the binding driven by the enthalpic contribution and the binding parameters just modulated by the presence of Mg^2+^. Finally, binding of FAD to *Spn*FADS in the presence of PPi could not be detected by enthalpic change, similarly to that occurred when titrating the PPi-free enzyme with oxidized FAD.

### *Spn*FADS exits mainly as a monomer but other oligomeric states are populated during catalysis

**Figure 3:**
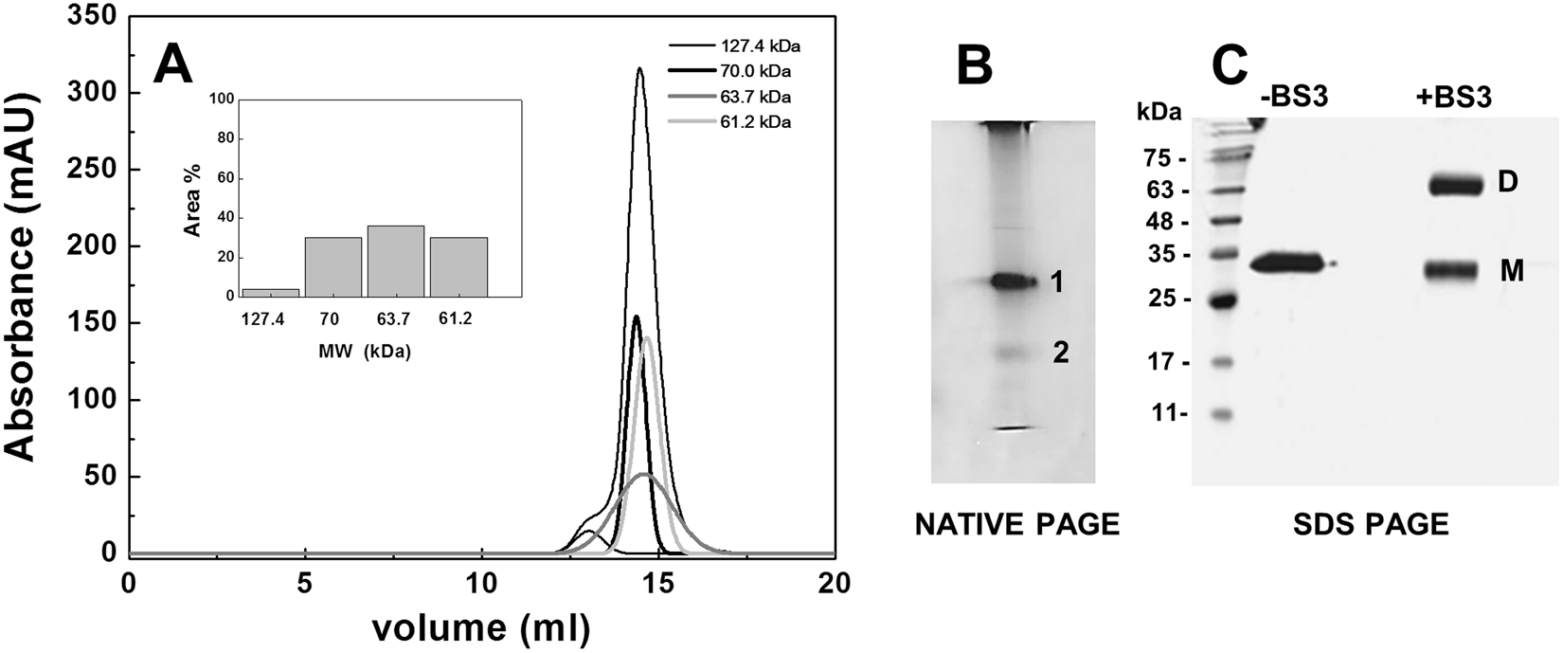
Hydrodynamic properties of *Spn*FADS. (**A**) Gel-filtration chromatogram of pure *Spn*FADS (20 µM) using a precalibrated Superdex^TM^ 200 10/300 GL column (*GE Healthcare*) equilibrated with 50 mM Tris/HCl, pH 8.0, 150 mM NaCl, and calibrated with the Gel Filtration Calibration Kit LMW (*GE Healthcare*). The inset shows the percentage of the fractions obtained from the chromatogram. (**B**) 12% Native PAGE of pure *Spn*FADS. The two major bands are denoted by number; 1 for predicted oligomers and 2, the most populated, for the putative monomer. (**C**) 15% SDS-PAGE of *Spn*FADS without crosslinker and after incubation with the BS3 crosslinker.

*Spn*FADS eluted in a main peak with a small shoulder in the gel filtration column used as a last step in its purification. The presence of the shoulder might be indicative of freshly purified *Spn*FADS stabilizing quaternary assemblies, as described for *Ca*FADS^1^. To check for such possibility we passed through the gel filtration column a freshly purified *Spn*FADS fraction considered as monomeric. This protein eluted as a main broad peak at ~14.5 mL (∼96% of the protein) but also produced a shoulder at ~13 mL (Fig. 3A). The peak at ~14.5 mL deconvolutes into three smaller peaks with apparent MWs of 61.2, 63.7 and 70 kDa. These values are slightly higher than the theoretic one for the monomer (34.8 kDa). The elongated structure of *Spn*FADS and the different conformations expected for its external loops could justify the reasonable assignment of those peaks to either different conformers of monomers or dimers. In contrast, the apparent MW of the shoulder at 13 mL is 127.4 kDa, suggesting this small protein fraction contains high MW oligomers. Pure preparations of *Spn*FADS also migrated in two bands on native gel electrophoresis, being one of them highly populated (band 1, Fig. 3B). According to the gel filtration data this band 1 can be putatively assigned to the monomer.

SDS-PAGE resolved the protein in a single band corresponding to the monomer MW, but pre-incubation of the protein with bis(sulfosuccinimidyl) suberate (BS3) crosslinker stabilized an additional band with an apparent MW of 78.6 kDa that can be assigned to a dimer (Fig. 3C). Altogether these data suggest that purified *Spn*FADS can be found in a monomer-dimer equilibrium that is mainly shifted towards the monomeric form, with traces of higher MW assemblies.

**Figure 4:**
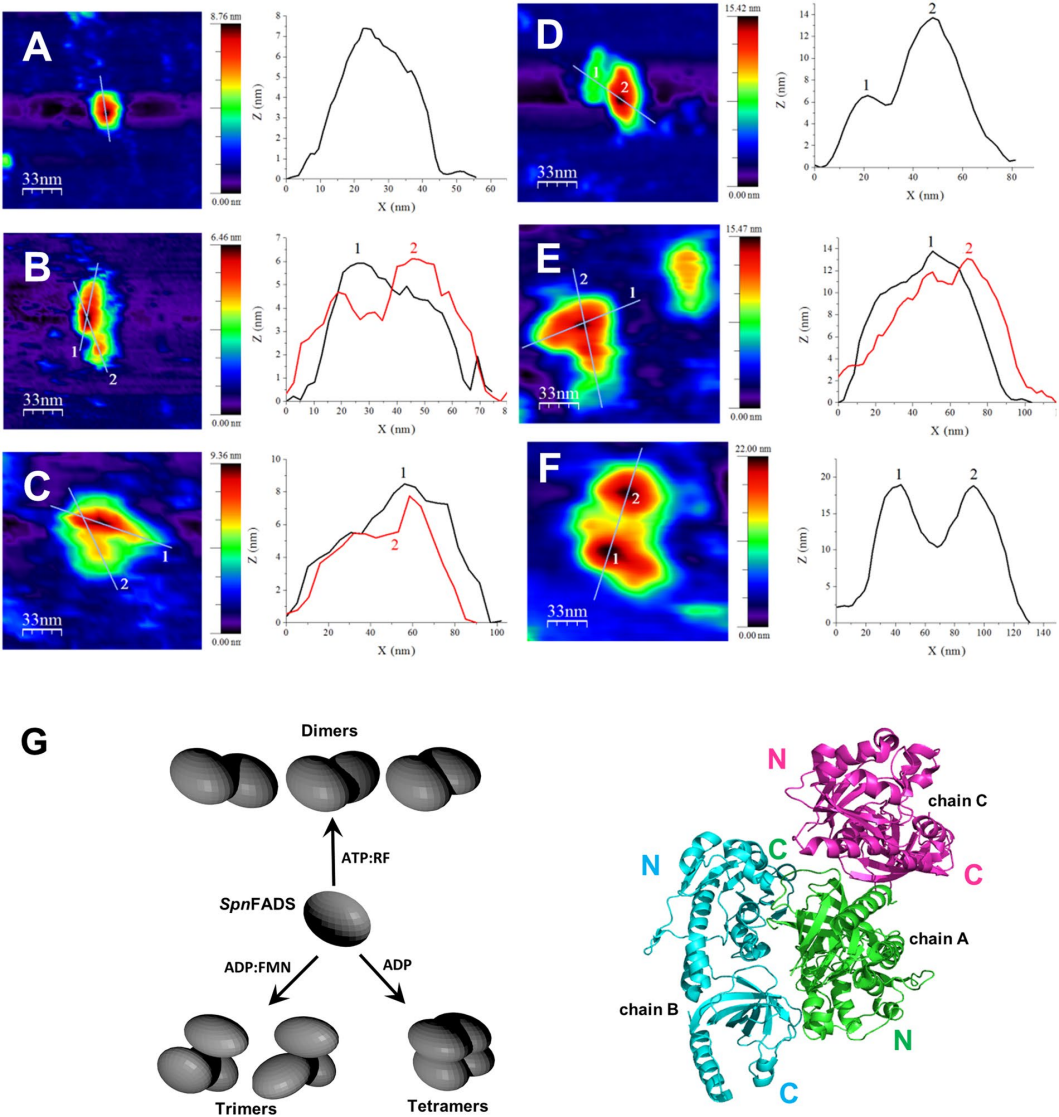
Image analysis of *Spn*FADS assemblies. Topography AFM images of a scanned area of 167 × 167 nm and Z-height profiles associated to the lines on the features of *Spn*FADS samples. (**A**) Monomer. (**B**) Dimer on the same plane. (**C**) Dimer with an overlapping on a monomer on the other. (**D**) Trimer from (**B**) showing an overlapped dimer interacting with a monomer. (**E**) Trimer from (**C**) showing overlapping of the dimer on a monomer. The different sizes of the original scanned images slightly change the roughness of the surface and the lateral resolution. This affects the size of the features in X-Y but not the Z-height measured in the profiles. (**F**) Tetramer. (**G**) Blender simulation of different assemblies according to the presence or absence of ligands and assembly of the three *Spn*FADS molecules in the crystal asymmetric unit (PDB ID: 3OP1).

We used atomic force microscopy (AFM) to shed light on the *Spn*FADS assembly in solution, as well as on the possible effect of ligands in the monomer-oligomer equilibrium (Fig. 4A–F). AFM topography images of free *Spn*FADS indicate a compact monomer (height ~7 ± 1 nm) (Fig. 4A). Incubation of the enzyme with different combinations of substrates and/or its reaction products produced some dimers and trimers, while ADP also promoted the formation of tetramers (Fig. 4 and Table 3). Two types of images were found for dimers. In one of them the two protomers dispose parallel in the same plane with the tallest module of one interacting protomer with the smallest of the other, leaving the rest of these molecules free of contact (Fig. 4B). In the other dimer, the most frequent, both protomers are on the same plane establishing different angles and slightly overlapping one module of the first onto one of the modules of the other (Fig. 4C). Both dimers appear as intermediate steps in the formation of the two trimeric species visualized in the images (Fig. 4D and E), which situate an additional monomer over one of the monomers of the dimeric structures. Considering the disposition adopted by the three molecules present in the asymmetric unit of the *Spn*FADS crystal (Fig. 4G), where each protomer is situated in a different plane, the trimers in solution differ from such crystallographic association. Finally, tetramers form two layers of two overlapped monomers (Fig. 4F). Incubation of *Spn*FADS with ATP and FMN, ADP and FMN, or FAD and PP_i_, produced the higher ratios of trimeric species, ~41%, while the RFK activity reactants, ATP and RF, generated 30% of dimeric molecules and only 16% of trimers. ATP by itself produced 37% of trimers, meanwhile ADP makes 20% of trimers and 47% of tetramers (Table 3). Incubation with the reactants of the FMNAT and FADpp activities resembles the same oligomeric yields. Since neither oxidized FMN nor FAD are able to bind to *Spn*FADS in the absence of adenine nucleotides, these data suggest that binding of adenine nucleotides (ATP and ADP) promotes the observed quaternary assemblies.

**Table 3:** Percentages of *Spn*FADS assemblies imaged by AFM.

Ligands	Units	ASSOCIATION STATE			
		Monomers (%)	Dimers (%)	Trimers (%)	Tetramers (%)
—	Features	97	3	—	—
	Molecules	94	6	—	—
ADP	Features	60	7	13	20
	Molecules	31	7	20	47
ATP	Features	70	12	18	—
	Molecules	47	16	37	—
ATP, RF	Features	73	20	7	—
	Molecules	54	30	16	—
ATP, FMN	Features	73	7	20	—
	Molecules	50	9	41	—
ADP, FMN	Features	63	15	22	—
	Molecules	40	19	41	—
FAD, PPi	Features	73	7	20	—
	Molecules	50	9	41	—

Samples of 0.5 μM *Spn*FADS were analyzed in 20 mM PIPES, pH 6.0, 2 mM DTT and 0.8 mM MgCl_2_ at room temperature. When indicated, ADP, ATP or PP_i_ were added at 250 μM, while oxidized FAD, FMN and RF were used at 50 μM. Features correspond to image units, while molecules refer to the amount of individual protein monomers in the corresponding image units. Error associated to percentage determination was ± 10%.

## Discussion

**Figure 5:**
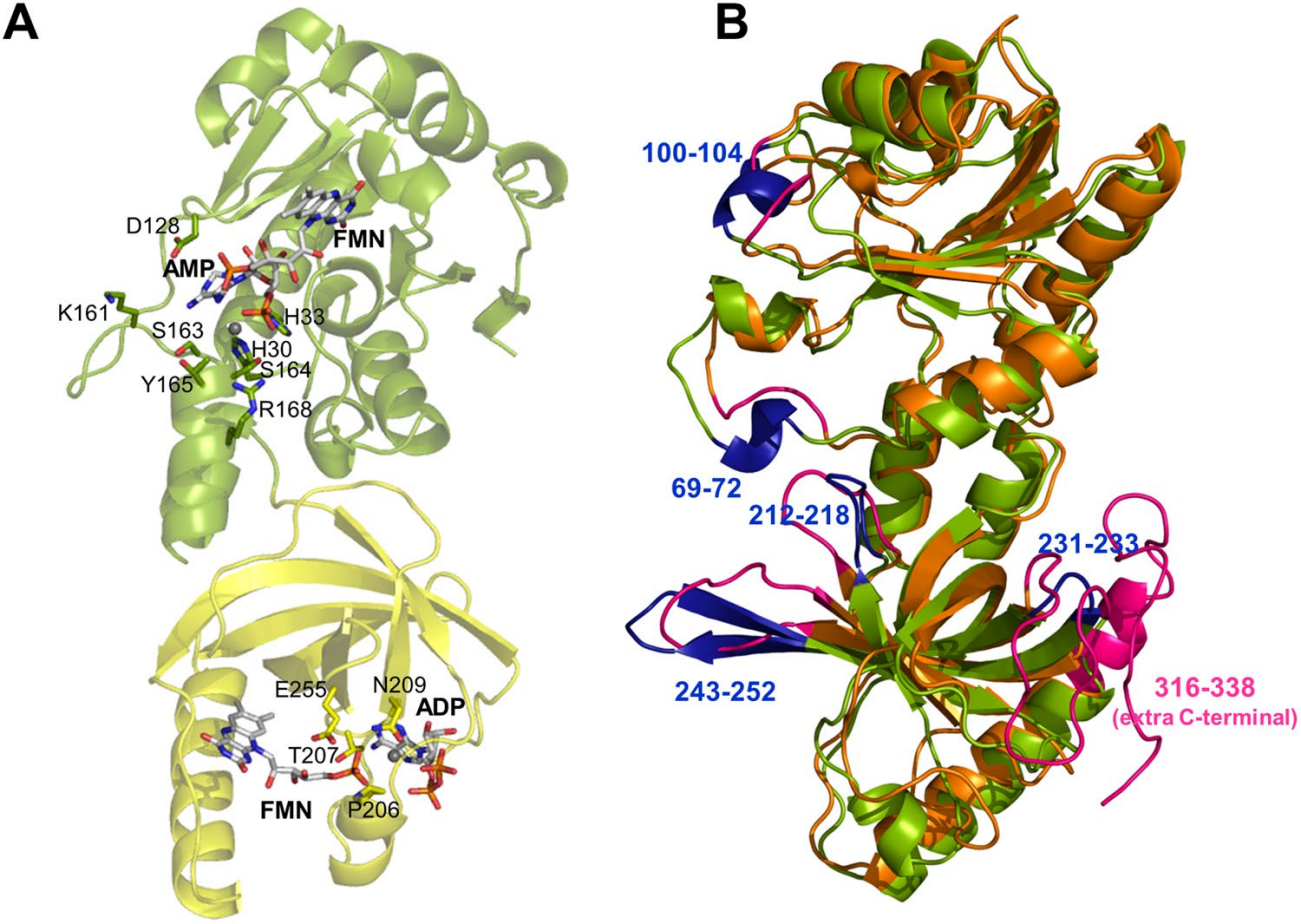
The structure of *Spn*FADS. (**A**) Model (based on PDB ID: 3OP1) for the interaction of *Spn*FADS with its ligands. The FMNAT module is shown in green and the RFK in yellow. Flavin and adenine nucleotide ligands are in CPK colored sticks with carbons in white. Mg^2+^ ions are shown as grey balls. Modelling performed using PyMol. (**B**) Superposition of the structures of *Spn*FADS (green, PDB ID: 3OP1) and *Ca*FADS (orange, PDB ID: 2X0K). Dissimilar regions are highlighted in blue and pink, respectively, including the numbers of implicated residues in *Spn*FADS.

Sequence alignments, structural comparisons^10, 13, 36^ and ligand binding models (Supplementary Fig. S1, and Fig. 5A) suggested that *Spn*FADS contains all the features of prokaryotic FADSs contributing to substrates stabilization and catalysis. Accordingly, we show here that the enzyme catalyzes the RFK, FMNAT and FADpp activities, but we also highlight different requirements for catalysis and binding of ligands relative to other family members. Interestingly while transformation of RF into FMN by *Spn*FADS occurs similarly under both aerobic and reducing conditions, the FMNAT and FADpp activities require reduced flavins (Fig. 1). Reducing conditions hardly influence any of the *Ca*FADS activities^5, 33^ (Table 1), but some bifunctional FADSs as well as the monofunctional *Hs*RFK (*Homo sapiens* RFK) are more active in the presence of high concentrations of reducing agents^34, 35, 42, 43^. Moreover, studies of enriched samples of the FADS from *Bacillus subtilis* (*Bs*FADS) early suggested specificity for reduced flavins in all its activities^4^.

Despite the differences in the redox requirements for the RFK and FMNAT activities between *Spn*FADS and *Ca*FADS, both enzymes showed the highest efficiency for the synthesis of FMN and FAD compared to the other family enzymes reported up to now (Table 1)^5, 33, 35, 42^, with the lack of inhibition by the RF substrate for the RFK activity in *Spn*FADS (Fig. 1C) as a notable difference between them. Our ITC data indicate that *Spn*FADS does not bind any of its oxidized flavinic substrates when adenine nucleotides are not present (Supplementary Fig. S4), contrary to *Ca*FADS^5, 33^. Reducing conditions are not a requirement for the binding of adenine nucleotides (ATP and ADP) (Fig. 2), and the presence of the adenine nucleotide also induces binding of oxidized RF or FMN. Nevertheless, such ternary interactions in *Spn*FADS are slightly weaker than in *Ca*FADS^1^. Moreover, in the presence of the adenine nucleotide, oxidized FMN only binds at one site, which according to the enzyme capability to transform oxidized RF into FMN must be the RFK site (Table 2). The fact that oxidized RF does not bind to free *Spn*FADS, the lack of inhibition by RF, and the enzyme being catalytically efficient at low RF concentrations, suggest a dissimilar RFK mechanism between this enzyme and *Ca*FADS^44^. Furthermore, the enzymatic and ligand binding characterizations of *Spn*FADS altogether confirm that competent flavin binding for catalysis at the FMNAT site requires the flavin ring in its reduced state. In addition, *Spn*FADS needs higher concentrations of substrates than *Ca*FADS to achieve maximal efficiency.

**Figure 6:**
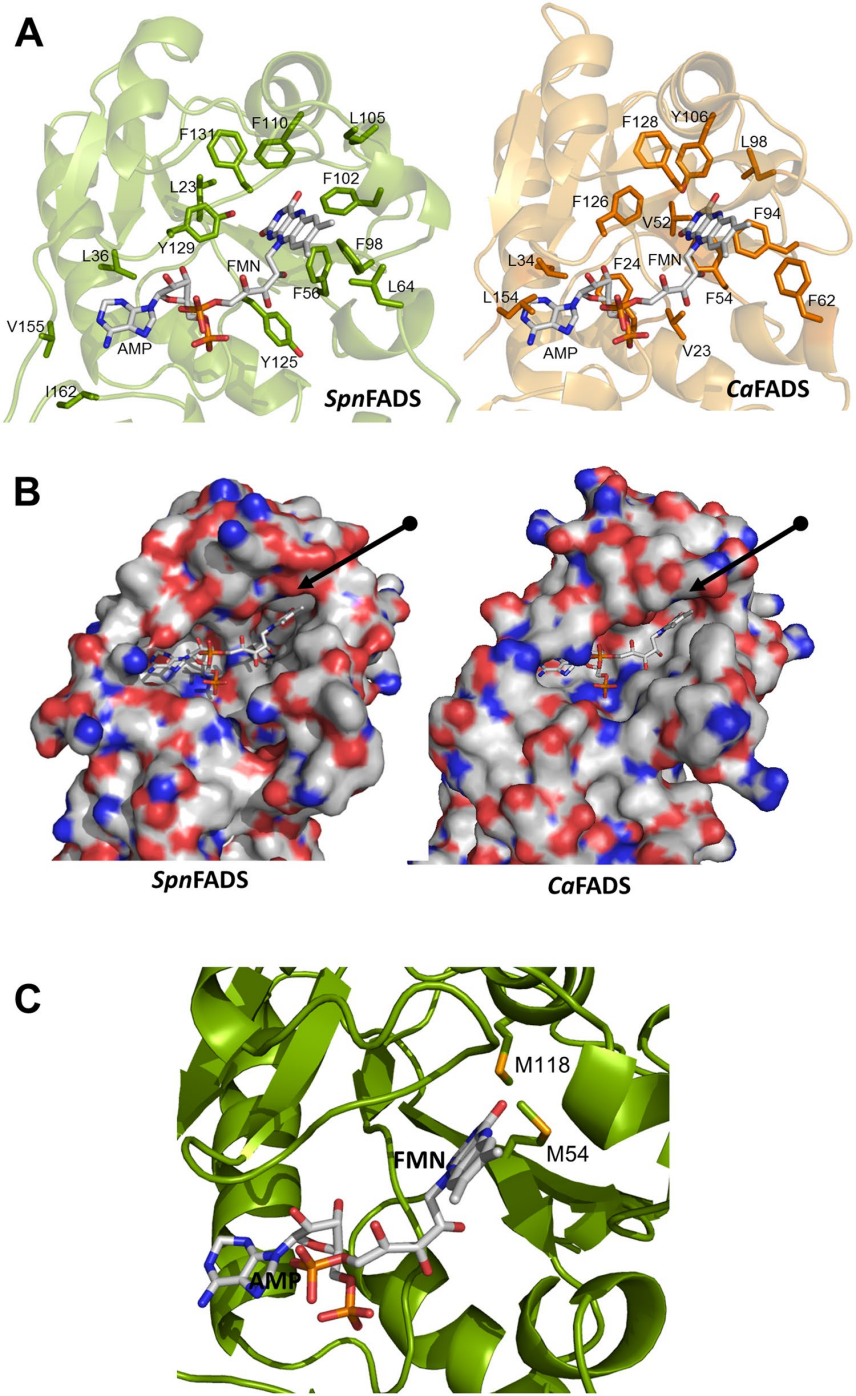
Comparative analysis of the *Spn*FADS and *Ca*FADS active sites in the FMNAT module. (**A**) Site for ligands binding in *Spn*FADS (green) and *Ca*FADS (orange). Hydrophobic and aromatic side chains are shown in sticks. (**B**) Surface electrostatic potential for the isoalloxazine binding cavity in *Spn*FADS (left panel) and *Ca*FADS (right panel). Arrows point to differences in sign of surface electrostatic potential at the *Spn*FADS and *Ca*FADS isoalloxazine sites. (**C**) Relative disposition of M54 and M118 regarding the isoalloxazine moiety of the flavin substrate. In all panels, FMN and AMP have been modelled on the crystals structures of *Spn*FADS (PDB ID: 3OP1) and *Ca*FADS (PDB ID: 2X0K) and represented in CPK sticks with carbons in grey^10^.

The need for reduced flavinic substrates in *Bs*FADS was interpreted as the geometry of the active site only allowing the incorporation of isoalloxazine rings in the reduced bended conformations, contrary to the highly stable planar organization adopted by their oxidized states^4, 17, 45, 46^. Despite the high conservation grade in overall folding (Supplementary Fig. S1) and key active sites residues, *Ca*FADS and *Spn*FADS are dissimilar in some regions involved in ligand binding (Fig. 5B and Supplementary Fig. S1). In the FMNAT module of *Spn*FADS helixes α3_10_n and α5n break loops L4n and L6n, respectively. These regions form the external part of the cavity where substrates for the FMNAT and FADpp activities bind. The sites for the ribityl and isoalloxazine moieties are formed in both enzymes by hydrophobic and aromatic side-chains (Fig. 6A), whose differential spatial position might determine cavity volumes and, therefore, modes for flavin binding. The cavity appears slightly broader and deeper in *Spn*FADS than in *Ca*FADS, although in *Spn*FADS the isoalloxazine binding site surface is rougher and shows a more negative surface electrostatic potential (Fig. 6B). The side chains of two Met residues, M54 and M118, form also part of the flavin ring cavity in *Spn*FADS, with M54 particularly prone to contact with the inner isoalloxazine ring (Fig. 6C). These Met residues are not conserved in most family members, which have instead hydrophobic residues^13^. Moreover, M54 forms part of a conserved sequence motif that is included only in few members such as *Bs*FADS.

**Figure 7:**
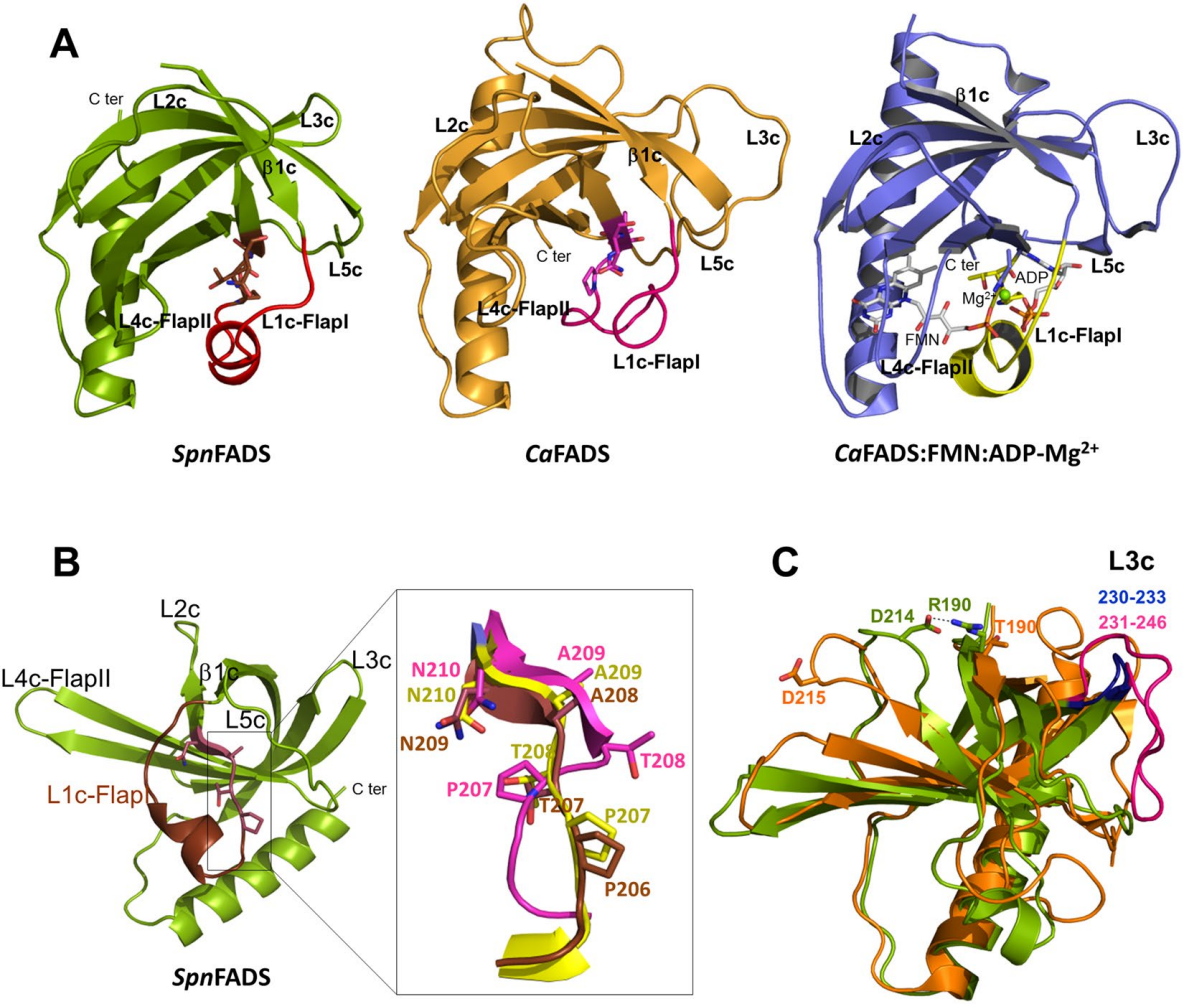
Comparative analysis of the RFK sites of *Spn*FADS, *Ca*FADS and the *Ca*FADS:FMN:ADP-Mg^2+^ ternary complex. (**A**) Cartoon representation of the RFK domains of *Spn*FADS (green, PDB ID: 3OP1), *Ca*FADS (orange, PDB ID: 2X0K) and ternary complex of the RFK module of *Ca*FADS (blue, PDB ID: 5A8A). L1c-FlapI is highlighted in red, pink and yellow, respectively. Residues of the PTAN motif are in CPK sticks. The FMN and ADP ligands bound to the *Ca*FADS RFK module are in white CPK and Mg^2+^ is included as a green sphere. (**B**) Detail of the PTAN motif in *Spn*FADS and superposition of the PTAN motives of the three structures. (**C**) Superposition of the RFK domain of *Spn*FADS (green) and *Ca*FADS (orange). The salt bridge between D214 at L2c and R190 at β1c of *Spn*FADS is in black dashes. Equivalent residues in *Ca*FADS, D215 and T190, are drawn in sticks. Loops L3c are colored in blue (*Spn*FADS) and pink (*Ca*FADS).

Structural differences are also observed when comparing the *Spn*FADS RFK module with the structures available for the *Ca*FADS RFK module free and in complex with ligands (Figs 5 and 7)^36^: i) D214 at loop L2c of *Spn*FADS salt bridges R190 at β1c; ii) loop L3c is 12 residues shorter; iii) the C-terminal is considerably shorter; and iv) an extra α helix, α1c, breaks loop L1c-Flap I. In addition, conformations of the conserved PTAN motif and of L1c-FlapI in free *Spn*FADS resemble those elements in the *Ca*FADS:FMN:ADP-Mg^2+^ complex. Such organization leaves the adenine nucleotide binding site open and the PTAN motif ready to stabilize the ligand (Fig. 7), contrary to free *Ca*FADS^36^. Thus, the weaker affinity of *Spn*FADS for the RFK activity products might be related with the lack of large conformational changes at the active site for substrates association and products dissociation during catalysis. These traits surely also contribute to the lack of inhibition by the RF substrate in *Spn*FADS.

Finally, substrates and products of *Spn*FADS induce formation of dimers, tetramers and particularly trimers with head-to-tail contacts between the RFK module and the FMNAT module of different protomers (Table 3, Fig. 4). None of these organizations resembles the *Ca*FADS dimer-of-trimers and in general the *Spn*FADS monomer appears to be majority^47^. The PDBePISA server predicts dimeric assemblies with head-to-tail fits (Supplementary Fig. S5 and Table S1), but it does not predict any higher oligomer as stable in solution. Differences in oligomerization profiles might relate with differences in protein oligomerization regions. In fact, a long L3c, only expected for *Corynebacterium* and *Mycobacterium* species, is predicted as a determinant to stabilize the dimer-of-trimers^47^. The shorter loop in *Spn*FADS agrees with this hypothesis, being less prone to oligomerization and establishing different contacts among protomers, which might also contribute to the dissimilar binding, kinetic and inhibition properties among species. The dimer-of-trimers in *Ca*FADS envisaged a controlled interplay between the regions involved in the formation of the RFK and/or FMNAT catalytic sites of different protomers as well as in flavin delivery to client apoflavoproteins^48, 49^. However, such mechanism might not be required for *Spn*FADS, since in this enzyme the main regulation mechanism for the FAD synthesis seems to be the reducing environment.

In conclusion, *Spn*FADS is a prokaryotic bifunctional FADS that folds in two modules, the C-terminal one being responsible for the RFK activity and the N-terminal one accounting for the FMNAT and FADpp activities. Three main characteristics distinguish *Spn*FADS from the previously well characterized *Ca*FADS: i) its FMNAT module only binds and transforms flavins in their reduced state, ii) its RFK activity does not exhibit inhibition by excess of RF substrate and iii) only dimers and trimers appear to be formed during catalysis. Considering the low number of members of the prokaryotic FADS family hitherto characterized, a large dissimilarity of optimal conditions for catalysis is envisaged. FADSs have a major functional role providing flavin cofactors to the cellular flavoproteome while also promoting flavins and flavoproteins homeostasis in all living organisms. In this context substrate inhibition, the use of reduced substrates or the formation of oligomeric assemblies might reflect different regulation mechanisms used by the enzyme to withstand relatively different environments. Such, differences among family members provide with a framework to the design of selective compounds targeting FADS for the treatment of diverse infectious diseases.

## Methods

### Cloning, Overexpression and Purification of *Spn*FADS

The DNA sequence encoding *Spn*FADS (accession number ADM84672, SPAP_1083), containing the *Nde*I and *Xho*I restriction sites and codons optimized for its expression in *E*. *coli*, was synthetically produced by Gen-Script. This DNA sequence was cloned into a modified pET-15b vector (*Novagen*) that contains an N-terminal His_6_-Tag sequence followed by a PreScission Protease site upstream of the *Nde*I and *Xho*I cloning sites. The final construct was fully sequenced at *Sistemas Genómicos* (www.sistemasgenomicos.com) and transformed into the *E*. *coli* strain Bl21 Star^TM^ (DE3) (*Invitrogen*). Transformed cells were grown at 37 °C in 2xTY medium (1.6% (*w*/*v*) tryptone, 1% (*w*/*v*) yeast extract, and 0.5% (*w*/*v*) NaCl) with 100 μg/mL of ampicillin until cells reached an OD_600_ of 0.6. Then the culture was cooled down to 18 °C and protein expression was induced by addition of 1 mM isopropyl β-D-1-thiogalactopyranoside. After 20 h of incubation at 18 °C, cells were harvested by centrifugation and suspended in 20 mM sodium phosphate, pH 7.4, 500 mM NaCl and 10 mM imidazole, containing lysozyme (1 mg/mL), DNase (0.1 mg/mL) and protease inhibitors (0.2 mM Phenylmethanesulfonyl fluoride (PMSF) and 10 mM benzamidine). After 30 min of incubation at 37 °C, cells were broken by sonication and centrifuged. The supernatant containing the soluble protein was loaded into a His-Trap affinity column (HisTrap HP, *GE Healthcare*) and the washing flow was discarded. Protein was eluted applying a gradient from 10 to 500 mM imidazole in 20 mM sodium phosphate, pH 7.4, 500 mM NaCl. Buffer was exchanged to 25 mM Tris/HCl, pH 7.4, 150 mM NaCl, using a HiPrep Desalting Column (*GE Healthcare*). The His_6_-Tag was removed by 48 h-incubation with PreScission protease (*GE Healthcare*) at 4 °C, in ratio 1:5 (*w*/*w*), and then the protein was loaded into the HisTrap HP and GSTrap 4B connected columns (*GE Healthcare*) to eliminate both the remained His_6_-Tagged *Spn*FADS and the PreScission protease (a GST fused product). The unbound fraction was recovered and further purified by size exclusion chromatography using a Superdex^TM^ 200 10/300 GL column (*GE Healthcare*) previously equilibrated with 20 mM PIPES, pH 7.0, 150 mM NaCl. Protein purity was assessed by 15% SDS-PAGE and NaCl was removed by dialysis in 25 mM Tris/HCl, pH 7.5 or 20 mM PIPES, pH 7.0 depending on later use. Pure protein solution was stored at −80 °C.

### Spectroscopic Analysis

UV-visible spectra were recorded in a Cary 100 spectrophotometer (*Varian*) and *Spn*FADS was quantified using the theoretical ε_279nm_ = 28.88 mM^−1^·cm^−1^ and a molecular weight of 34,521 Da (ProtParam). Circular dichroism (CD) spectra were recorded with a Chirascan spectropolarimeter (*Applied Photophysics Ltd*.) at 25 °C. Samples containing ∼5 and ∼20 µM *Spn*FADS in 25 mM Tris/HCl, pH 7.5 were used in the far-UV (cuvette path length, 0.1 cm) and near-UV CD (cuvette path length, 0.4 cm), respectively.

### Qualitative and quantitative analysis of the activities of *Spn*FADS

RFK, FMNAT and FADpp activities of *Spn*FADS were qualitatively assayed by separating the different flavins present in the reaction mixtures using thin layer chromatography on Silica Gel SIL-G-25 plates as previously described^13^. Reaction mixtures containing 50 μM flavin (either RF, FMN or FAD), 0.2 mM ATP or PP_i_, 0.8 or 10 mM MgCl_2_, and 0.5 μM *Spn*FADS in 150 μL of 50 mM Tris/HCl, pH 8.0, were incubated in the presence of different sodium dithionite concentrations (0–16 mM) at 25 °C during 30 min. The reaction was stopped by boiling the samples at 100 °C for 5 min. Flavin mixtures containing 50 µM of each flavin, RF, FMN and FAD, were included as standards. Flavin TLC spots were examined by monitoring their fluorescence under UV light.

RFK and FMNAT activities of *Spn*FADS were quantitatively measured at 25 °C in 20 mM PIPES, pH 7.0, 0–10 mM MgCl_2_ as previously described^5, 33^, and when indicated 0–10 mM sodium dithionite was also added. Reaction mixtures (total volume 500 μL) containing 0.5–15 µM RF or 1–55 µM FMN, and 10–400 µM ATP were pre-incubated at 25 °C until reaction was initiated by addition of ∼25 nM enzyme. In all cases, reaction was stopped after 1 min of incubation through protein denaturation by boiling the sample for 5 min. Precipitated protein was removed from the solution by centrifugation at 18,000 x g during 10 min. The flavin composition of supernatants was analyzed using an Alliance HPLC system (*Waters*) equipped with a 2707 autosampler and an HSST3 column (4.6 × 150 mm, 3.5 µm, *Waters*) preceded by a precolumn (4.6 × 20 mm, 3.5 µm, *Waters*) as previously described^5, 33^. The produced amounts of FMN or FAD were quantified using their corresponding standard curves acquired under the same conditions. The kinetic data obtained for one substrate at saturating concentrations of the second substrate (as nmol of flavin transformed *per* min) were interpreted using the Michaelis-Menten kinetic model, obtaining *k*_cat_ and *K*_m_ values with errors of ±10%. Controls for transformations of flavins in the absence of the enzyme under all reaction conditions showed only negligible processes.

Additional experiments were performed to elucidate whether reduced flavins are required for the FMNAT activity, or if the reducing environment is a requirement for the enzyme. Reaction mixtures containing 500 μM ATP, 15 μM FMN, 2 μM 5-deazaRF (5-dRF) and 0.8 mM MgCl_2_ in a final volume of 1 mL were deoxygenated during 1 h through Argon bubbling in the dark (to avoid FMN degradation). Once oxygen was displaced from the samples, previously deoxygenated *Spn*FADS was added at a final concentration of 100 nM. Then samples were illuminated during 15 s to photo-reduce FMN (the triplet state of 5-dRF, obtained by light-excitation, quickly evolves to a radical state that returns to the fundamental state by donating an electron to FMN). After 3 min of additional incubation samples were boiled for 5 min (to stop the reaction) and then centrifuged. Flavins present in the supernatant were identified by HPLC as we indicated above.

### High-Sensitivity Isothermal Titration Calorimetry (ITC)

ITC measurements were performed using an AutoITC200 calorimeter (*MicroCal-Malvern*) thermostated at 20 °C following the procedures previously reported^5, 33^. Typically, 300 μM nucleotide (ATP/ADP) or 150–200 μM flavin (RF/FMN/FAD) solutions were used to titrate ~20 μM *Spn*FADS solutions. When appropriate, ternary titrations were performed by adding flavin solutions to a mixture of the protein pre-incubated with 400 μM ADP. Ligands and *Spn*FADS were dissolved in 20 mM PIPES pH 7.0, with 0, 0.8 or 10 mM MgCl_2_, and degassed prior to titration. Up to 19 injections of 2 µL were programmed with enough time spacing for the signal to recover the baseline. Similar protein concentrations in the calorimetric cell were employed in all experiments to guarantee that the oligomerization state of the protein is the same at any time. The association constant (*K*_a_), the enthalpy change (Δ*H*) and the stoichiometry (N) were estimated through non-linear regression of the experimental data using a model for one or two independent binding sites; the regression was implemented in Origin 7.0 (*OriginLab*). The dissociation constant (*K*_d_), the free energy change (Δ*G*), and the entropy change (Δ*S*) were obtained from basic thermodynamic relationships. Errors considered in the measured parameters ( ± 20% in *K*_d_ and ± 0.3 kcal/mol in Δ*H* and –TΔ*S*) were taken larger than the standard deviation between replicates and the numerical error after the fitting analysis.

Titrations performed to elucidate whether *Spn*FADS was able to bind reduced FMN and FAD were performed using a high precision VP-ITC system (*MicroCal LLC*), and experimental sets were similar to those for the experiments in the AutoITC200 calorimeter. Thus, 25 μM *Spn*FADS were titrated at 20 °C with FMN ≈ 200 μM or FAD ≈ 300 μM. Both the protein and the ligands were dissolved in 20 mM PIPES, pH 7.0, 0.8 mM MgCl_2_, 4 mM sodium dithionite, 10 mM glucose, 0.5 U/mL glucose oxidase, and degassed. Each titration was initiated by a 4 μL injection followed by 25–28 stepwise injections of 10 μL. Glucose and glucose oxidase were added to avoid oxidation of sodium dithionite by molecular oxygen in order to avoid unspecific oxidation heat that could mask the one corresponding to the flavin:protein interaction.

### Evaluation of the hydrodynamic and quaternary organization properties

Stabilization of the quaternary organizations of *Spn*FADS was initially evaluated by gel filtration onto a Superdex^TM^ 200 10/300 GL column (*GE Healthcare*), equilibrated with 50 mM Tris/HCl, pH 8.0, 150 mM NaCl, and calibrated with the Gel Filtration Calibration Kit LMW (*GE Healthcare*)^47^. Oligomer formation was also analyzed by incubation of a pure fraction of *Spn*FADS in 50 mM KHPO_4_, pH 8.0 with BS3 crosslinker (*Thermo Scientific*) for 2 h at 4 °C. The cross reaction was stopped adding the quenching solution (Tris/HCl 500 mM (10x), pH 8.0) up to 50 mM and the different oligomeric states of the protein were visualized on a 15% SDS-PAGE gel.

For single molecule visualization of *Spn*FADS preparations, atomic force microscopy imaging was performed using a Multimode 8 AFM system (*Bruker*). Images were taken using the Tapping Mode with calibrated soft V-shaped silicon nitride cantilevers carrying pyramidal 2 nm ultrasharp tips characterized by spring constants ranged from 0.01 to 0.03 N/m and nominal resonance frequencies of 7 to 15 kHz (*Bruker Probes*). For samples containing ligands, the mixtures were pre-incubated at 4 °C for 10 min under mild stirring to favor complex formation prior to immobilization^47^. ADP, ATP and PP_i_ were added at 250 µM, while FAD, FMN and RF were used at 50 µM. *Spn*FADS solutions (∼0.5 μM) were immobilized on small fresh exfoliated muscovite mica pieces (*Electron Microscopy Sciences*) for 10 min at room temperature. The sample and holder were introduced in a liquid cell previously cleaned with 20% isopropanol and Millipore ultrapure water. AFM measurements were conducted in 20 mM PIPES, pH 6.0, freshly prepared 2 mM dithiothreitol (DTT, *Sigma-Aldrich*) and 0.8 mM MgCl_2_ at room temperature. Raw images were processed using the WSxM freeware^50^. Percentages of different features were calculated taking several sample areas with several scan sizes, zooming with the WSxM software functions without losing relevant information and discarding artifacts^51^. A considerable amount of features were collected for the statistical population study. Total protein percentages were also calculated considering the number of molecules involved in each type of association. Errors were calculated from the dispersion of results in the analysis of different AFM images corresponding to different areas of the sample.

### Structural prediction of enzyme-ligand interactions

Models for ligand binding at the RFK and FMNAT sites of *Spn*FADS were produced comparing its structure (chain B of the crystal structure PDB ID: 3OP1) with the previously reported *Ca*FADS crystal structures (for the RFK module) or models (FMNAT module) in complex with their oxidized substrates^10, 36^. The PyMol 0.99^52^ software was used for complex modelling, structural analysis and figures production.

### Data availability statement

All data generated or analyzed during this study are included in this published article (and in its Supplementary Information files).

## Electronic supplementary material

Supplementary Material
