# Supplementary material for: The FAD synthetase from the human pathogen Streptococcus pneumoniae: a bifunctional enzyme exhibiting activity-dependent redox requirements
Source: Sci Rep. 2017 Aug 8;7:7609. doi: 10.1038/s41598-017-07716-5 (PMC5548840; doi:10.1038/s41598-017-07716-5)
Supplement: Supplementary file 1 — Supplementary Material [file 41598_2017_7716_MOESM1_ESM.pdf]

Supplementary Material

The FAD synthetase from the human pathogen *Streptococcus pneumoniae*: a bifunctional enzyme exhibiting activity-dependent redox requirements

María Sebastián[1,2,#], Erandi Lira-Navarrete[2,6,#], Ana Serrano [1,2,7], Carlos Marcuello[3,8], Adrián Velázquez-Campoy[1,2,4,5], Anabel Lostao[3,4], Ramón Hurtado-Guerrero[2,4], Milagros Medina[1,2,]*, and Marta Martínez-Júlvez[1,2,]*

[1] Departamento de Bioquímica y Biología Molecular y Celular, Facultad de Ciencias, Universidad de Zaragoza, Spain

[2] Instituto de Biocomputación y Física de Sistemas Complejos (BIFI) and GBsC-CSIC and BIFI Joint Units, Universidad de Zaragoza, Spain

[3] Laboratorio de Microscopías Avanzadas (LMA), Instituto de Nanociencia de Aragón (INA), and Fundación INA, Universidad de Zaragoza, Spain

[4] Fundación ARAID, Diputación General de Aragón, Spain

[5] Aragon Institute for Health Research (IIS Aragon), Zaragoza, 50009, Spain

[#] These two authors contributed equally to this work.

*Correspondence to: Marta Martínez-Júlvez. Departamento de Bioquímica y Biología Molecular y Celular. Facultad de Ciencias. Pedro Cerbuna 12. Universidad de Zaragoza. 50009-Zaragoza. Spain. Fax: +34 976 762123; Phone: +34 976 762841 e-mail: mmartine@unizar.es. Milagros Medina. Departamento de Bioquímica y Biología Molecular y Celular. Facultad de Ciencias. Pedro Cerbuna 12. Universidad de Zaragoza. 50009-Zaragoza. Spain. Fax: +34 976 762123; Phone: +34 976 762476 e-mail: mmedina@unizar.es

Current addresses: [6] Copenhagen Center for Glycomics, Department of Cellular and Molecular Medicine, School of Dentistry, University of Copenhagen, Copenhagen DK-2200, Denmark; [7] Centro de Investigaciones Biológicas, CSIC, Ramiro de Maeztu 9, E-28040 Madrid, Spain; [8] Univ Reims, Lab Rech Nanosci, EA4682, F-51100 Reims and INRA, FARE Lab F-51100 Reims, France.

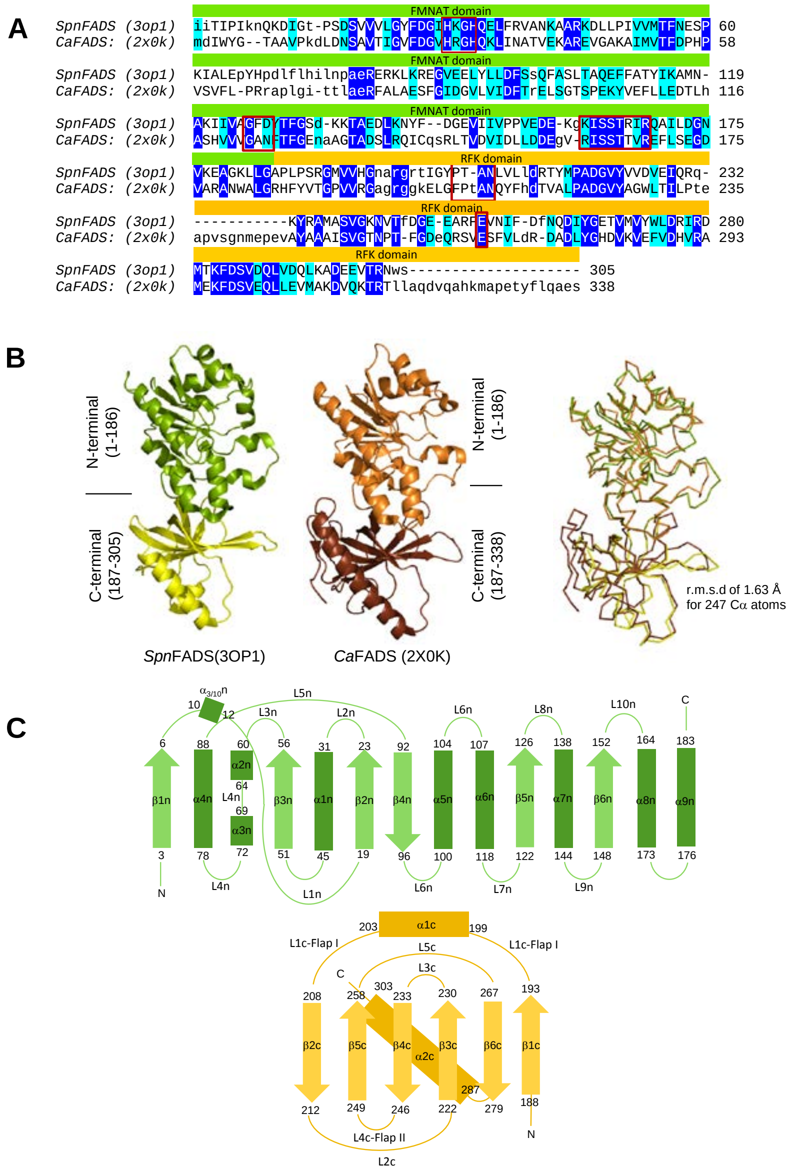

Figure S1: Structural properties of *Spn*FADS. **(**A) Structural alignment of *Spn*FADS (PDB 3OP1) and *Ca*FADS (PDB 2X0K) according to the SSM server (http://www.ebi.ac.uk/msd-srv/ssm/). Identical residues are shown in dark blue and conservative ones in light blue. Consensus motifs are highlighted with boxes. *Spn*FADS contains the 30-**HKGH**-33, 126-GF**D-**128 and 161-**KISST**RI**R**-168 consensus motifs in the N-terminal module contributing to ATP stabilization for FMN adenylylation, as well as in FAD stabilization for the FADpp activity. The enzyme also conserves the 206-**PTAN**-209 motif and E254 at the RFK module to set up the catalytic site to transform RF into FMN. (*Spn*FADS numbering, highly conserved residues along family are shown in bold). (B) Three dimensional structures of *Spn*FADS (N- and C-terminal modules colored in green and yellow, respectively) and *Ca*FADS (orange and brown, respectively). On the right it is shown the superposition of both structures showing an r.m.s.d. of 1.63 Å for 247 Cα atoms (structural alignment by PyMol). (C) Topology of the N-terminal FMNAT module (green) and the C-terminal RFK module (orange) of *Spn*FADS. α helices are shown as solid dark rectangles and β strands as light arrows. Numbers indicates residue positions.

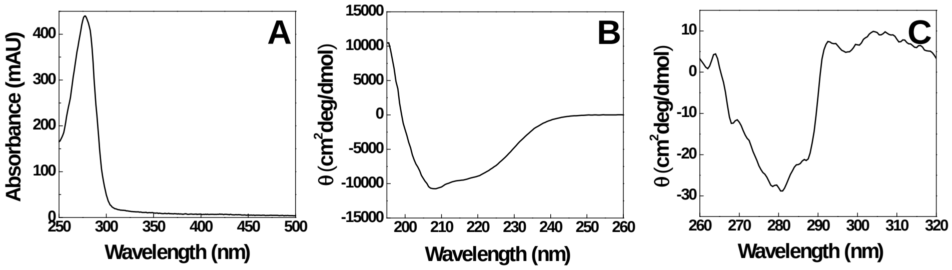

Figure S2: Spectroscopic properties of *Spn*FADS. (A) UV-visible absorption spectrum of *Spn*FADS (15 µM). (B) Far-UV circular dichroism spectrum of *Spn*FADS (5 µM). (C) Near-UV circular dichroism spectrum of *Spn*FADS (20 µM). Spectroscopic measurements were performed in 25 mM Tris/HCl, pH 7.5 at room temperature.

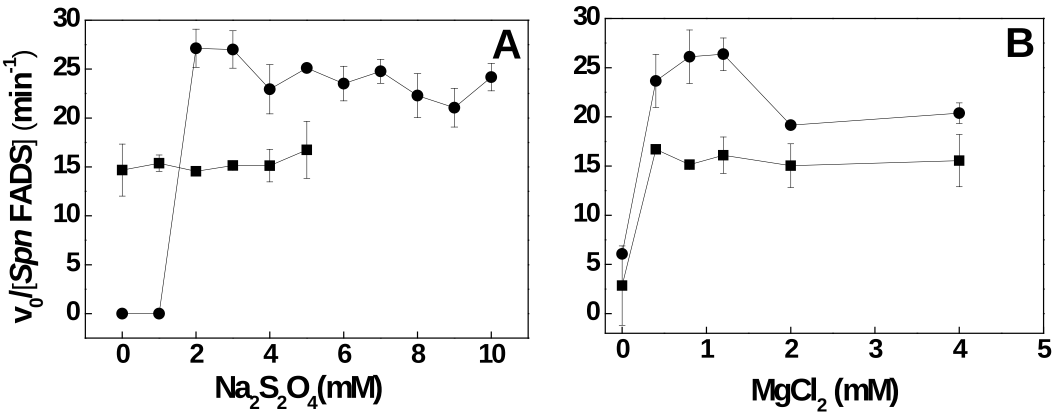

Figure S3: Rates of the RFK (■) and FMNAT (●) activities of *Spn*FADS as a function of (A) sodium dithionite and (B) $MgCl_2$ concentrations. Reaction mixtures contained ATP (~240 µM), RF (6 µM) and *Spn*FADS (25 nM) for the RFK activity; ATP (~250 µM), FMN (20µM) and *Spn*FADS (25 nM) for the FMNAT activity. In all assays $MgCl_2$ was used at 0.8 mM when varying sodium dithionite, while sodium dithionite was used at 3 mM when varying $MgCl_2$.

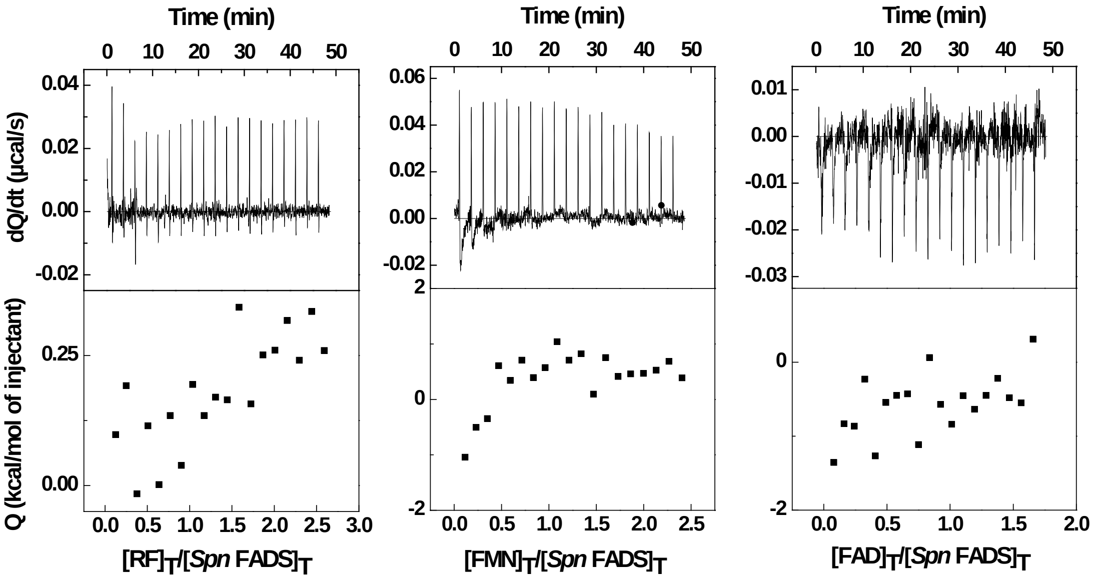

Figure S4: Representative calorimetric titrations of the interactions between *Spn*FADS with flavin cofactors: RF, FMN and FAD. The upper panels show the thermograms for the interactions and the lower panels show the corresponding binding isotherms with integrated heats. Experiments were carried out in 20 mM PIPES, pH 7.0 in 10 mM $MgCl_2$ at 20 °C and indicated flavins were not binding under these conditions.

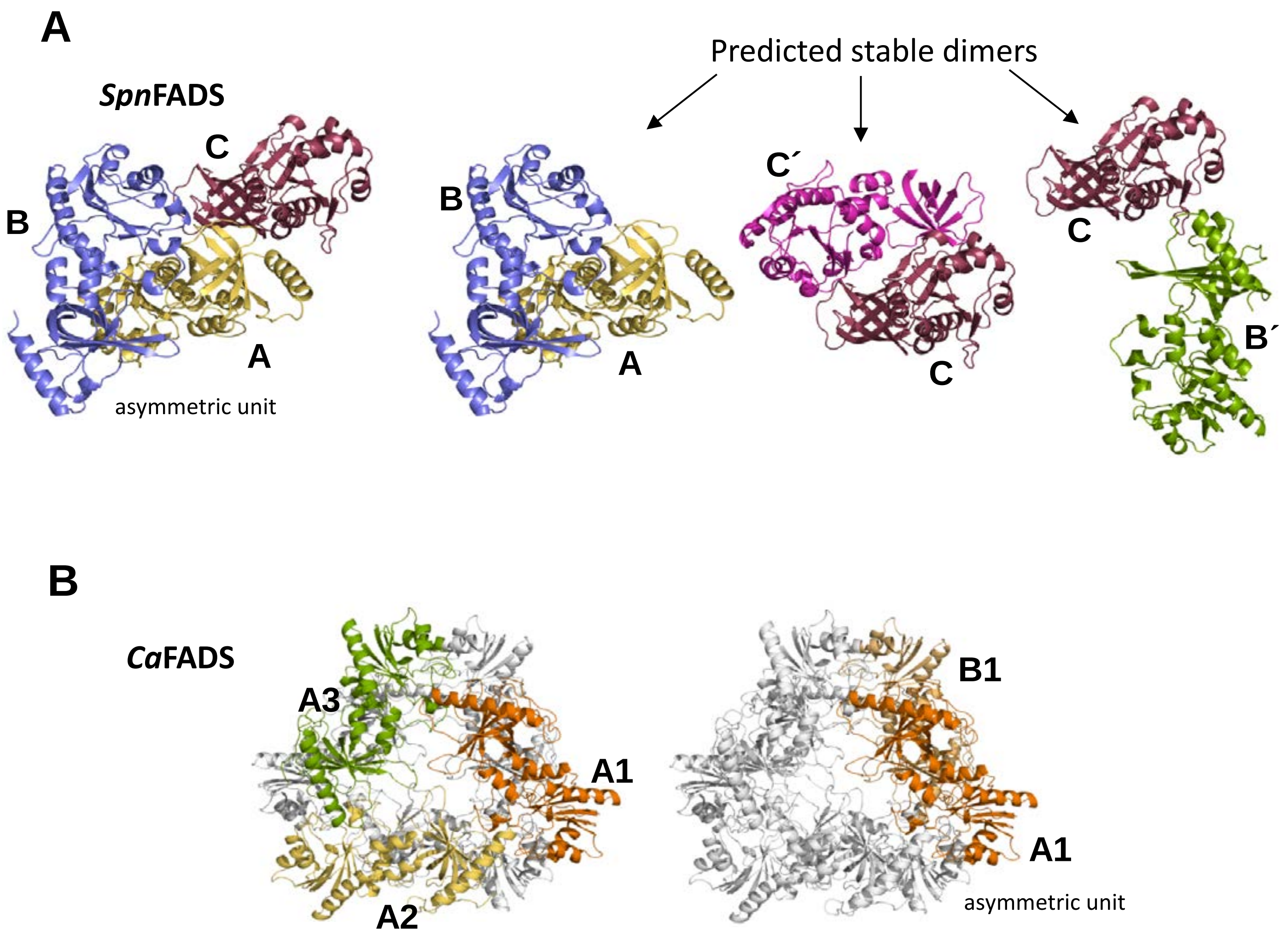

Table SP1.- List of probable quaternary structures in solution for *Spn*FADS and *Ca*FADs as predicted by the PISA server

Composition	Stable	Surface area Å^2	Buried area Å^2	ΔG^{int} kcal/mol	ΔG^{diss} kcal/mol
***Spn*FADS**					
CC´	yes	24440	7030	-82.0	9.2
AB	yes	28530	5090	-33.2	2.9
B´C	yes	28190	4580	-52.6	0.3
***Ca*FADS**					
A_3B_3	yes	82800	19770	-189.0	22.5
B_3	yes	45020	6550	-73.0	3.1
A_3	yes	44480	6520	-72.0	2.7
AB	yes	31390	2800	-52.7	12.4

Figure S5: (A) Oligomeric organizations predicted by the PDBePISA server based on the structure of *Spn*FADS (PDB 3OP1). The asymmetric unit contains three chains, A, B, and C, coloured in yellow, blue and red, respectively. Besides, the three predicted as possible stable dimers in solution. Chains A´ (pink) and C´ (green) belong to other asymmetry unit. (B) Oligomeric assembly predicted for *Ca*FADS as a dimer-of-trimers (hexamer, A_3B_3). The left panes shows the protomers of the trimer at front coloured in orange, green and yellow and the others at the back in grey. The right panels show the location of the two chains of asymmetric unit (A_1B_1) in the dimer-of-trimers (orange and pale orange).
